# Oxidative status in dairy goats: periparturient variation and changes in subclinical hyperketonemia and hypocalcemia

**DOI:** 10.1186/s12917-021-02947-1

**Published:** 2021-07-06

**Authors:** Yan Huang, Jing Wen, Yezi Kong, Chenxu Zhao, Siqi Liu, Yaoquan Liu, Lan Li, Jiaqi Yang, Xiaoyan Zhu, Baoyu Zhao, Binyun Cao, Jianguo Wang

**Affiliations:** 1 College of Veterinary Medicine, Northwest A&F University, 712100 Yangling, Shaanxi China; 2 College of Animal Science and Technology, Northwest A&F University, 712100 Yangling, Shaanxi China

**Keywords:** Peripartum, Energy deficit, Redox status, Ketone body, Calcium

## Abstract

**Background:**

A better comprehension of the redox status during the periparturient period may facilitate the development of management and nutritional solutions to prevent subclinical hyperketonemia (SCHK) and subclinical hypocalcemia (SCHC) in dairy goats. We aimed to evaluate the variation in the redox status of dairy goats with SCHK and SCHC during their periparturient periods. Guanzhong dairy goats (n = 30) were assigned to SCHK (n = 10), SCHC (n = 10), and healthy (HEAL, n = 10) groups based on their blood β-hydroxybutyrate (BHBA) and calcium (Ca) concentrations. Blood were withdrawn from goats every week from 3 weeks before the expected parturition date to 3 weeks post-kidding. On the same day, the body condition scores (BCS) were evaluated, and the milk yield was recorded for each goat. The metabolic profile parameters and the indicators of oxidative status were determined by using the standard biochemical techniques.

**Results:**

In comparison with the HEAL goats, SCHK and SCHC goats presented with a more dramatic decline of BCS post-kidding and a significant decrease in the milk yield at 2- and 3-weeks postpartum, ignoring the obvious increase at 1-week postpartum. The levels of non-esterified fatty acids (NEFA) peaked at parturition, exhibiting significantly higher levels from 1-week prepartum to the parturition day in the SCHK and SCHC groups. The malondialdehyde (MDA) concentration was increased in the SCHK goats from 1-week antepartum until 3-weeks postpartum, with its concentration being significantly higher in the SCHC goats at parturition. The hydrogen peroxide (H_2_O_2_) concentration was significantly lower in the SCHK and SCHC goats from 2-weeks antepartum to 1-week post-kidding. The total antioxidant capacity (T-AOC) and the superoxide dismutase (SOD) level were decreased at 1-week antepartum in the SCHK and SCHC goats, respectively. The glutathione peroxidase (GSH-Px) level was increased in the SCHK and SCHC goats during the early lactation period.

**Conclusions:**

The SCHK and SCHC goats exerted more efforts to maintain their redox homeostasis and to ensure the production performance than the HEAL goats during their periparturient period, probably owing to more intense fat mobilization and lipid peroxidation in the former.

## Background

The periparturient period is extremely crucial with respect to maintaining the health and productive performance of dairy goats. During this period, the dry matter intake (DMI) often cannot meet the nutrient demands necessary for healthy fetal growth and lactogenesis, resulting in an energy deficit [1, 2]. Under this condition of metabolic adaptations corresponding to energy deficit, the catabolic pathways of adipose tissues can be triggered; subsequently, the increase in the systemic generation of reactive oxygen species (ROS) that react with every organic molecule they encounter to produce reactive oxygen metabolites (ROMs), including superoxide anions (O_2_^−^), hydroxyl radicals (·OH), and hydrogen peroxide (H_2_O_2_) [3–5]. Malondialdehyde (MDA), an end-product of ROS-induced lipoperoxidation, can induce oxidative damage and cytotoxicity by attacking cellular macromolecules (such as proteins and nucleic acids) [6]. To counteract this oxidative damage, the organisms are equipped with endogenous antioxidants as a scavenging system. However, oxidative stress occurs in case of a damage to the cellular macromolecules or in case of tissue dysfunction [7]. During the late pregnancy and early lactation period, the dairy stock experiences multiple challenges to maintaining homeostasis imposed by significant physiological and metabolic stressors, which, in turn, trigger production diseases such as ketosis and hypocalcemia [8]. Accumulating evidence suggests that oxidative stress influences the pathogenesis of several metabolic disorders throughout the transition period [9–12].

Subclinical hyperketonemia (SCHC) and subclinical hypocalcemia (SCHC) are common metabolic disorders that occur during the periparturient period in intensive dairy farms [13, 14], although these diseases usually present with no specific signs distinct from other clinical forms of diseases. However, dairy cows with SHCK and SCHC are more susceptible to other peripartal diseases, such as mastitis and retained fetal membranes [15, 16]. The study on SCHK and SCHC goats with health disorders during the transition period is scarce, but it probably shares some similarity with the situation of cows. The prevalence of SCHK and SCHC in China is 10 and 22 % [17], respectively, which has contributed to the economic losses to the dairy goat industry because of the resultant decrease in milk production and the veterinary expense incurred in association with the care and treatment of goats afflicted with SCHK and SCHC-associated periparturient diseases. Oxidative stress can alter the physiology and cause pathologies [9]. Some past studies have reported that hyperketonemia in dairy cows and buffaloes is closely associated with the elevation of the oxidative status levels [18–20]. Furthermore, other past reports have presented a variation in the oxidative status levels during the periparturient periods of sheep and dairy goats [21, 22]. However, the redox state of SCHK and SCHC in dairy goats during this period remains unclear.

A better understanding of the redox status during the transition period may facilitate the development of management and nutritional solutions to prevent metabolic disorders such as SCHK and SCHC in dairy goats. In the present study, we investigated the variation in the oxidative status in dairy goats with SCHK and SCHC and in healthy (HEAL) goats during the periparturient period by evaluating the levels of metabolic parameters and the oxidative status-related indexes in the plasma.

## Results

### BCS and milk production

**Fig. 1:**
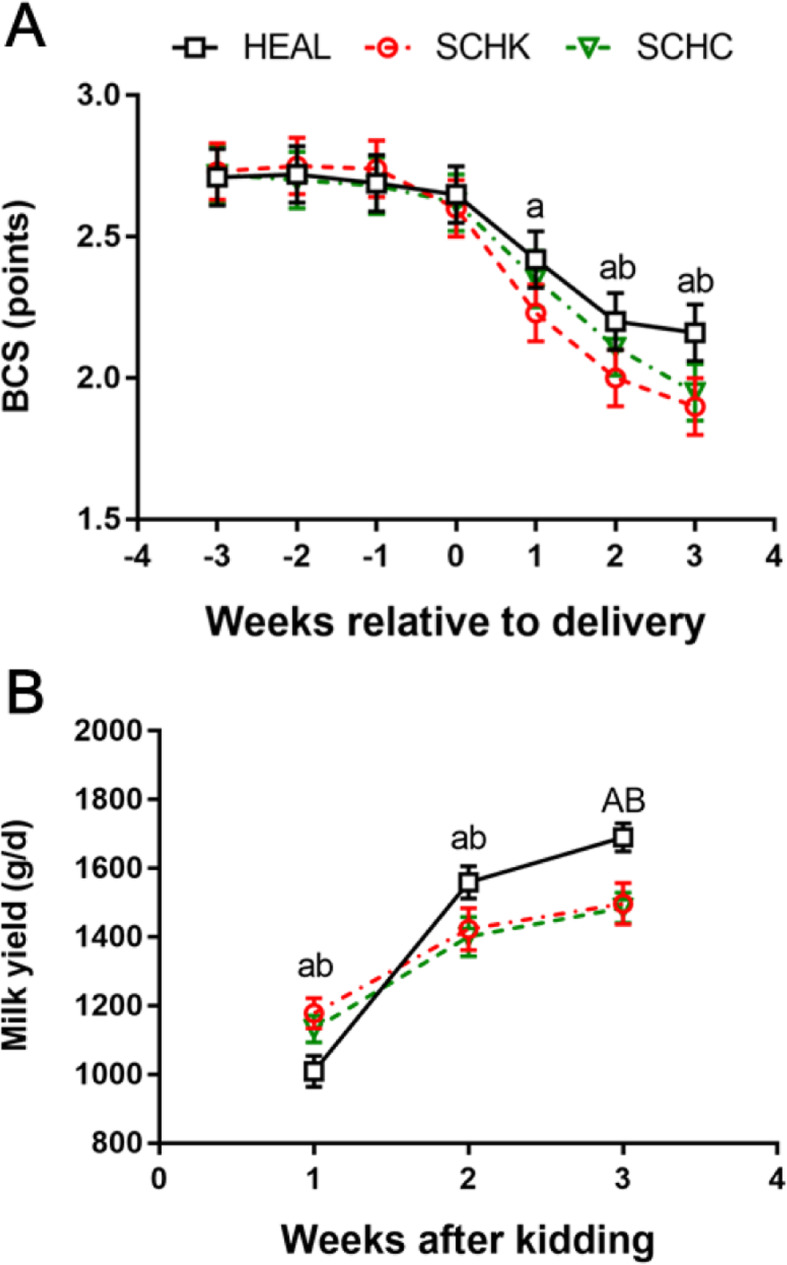
Time-course of BCS (**A**) and milk yield (**B**) in healthy (HEAL), subclinical hyperketonemia (SCHK), and subclinical hypocalcemia (SCHC) goats. Different line forms represent the individual groups included in the study. BCS and milk yield were analyzed using repeated measurements ANOVA, followed by Tukey’s multiple comparisons test. Repeated measures for each goat were considered (repeated factor: time during the periparturient period). ^A^*P* < 0.01 and ^a^*P* < 0.05 between the SCHK and HEAL groups. ^B^*P* < 0.01 and ^b^*P* < 0.05 between the SCHC and HEAL groups. Values are expressed as means ± SEM from 10 goats/group (*n* = 10)

During the postpartum weeks 1–3, all the groups demonstrated a decline in the body condition scores (BCS; Fig. 1 A). In comparison with the HEAL goats, BCS was significantly decreased from 1- to 3-weeks postpartum in SCHK goats (*P* < 0.05), and at 2- and 3-weeks postpartum in SCHC goats (*P* < 0.05). The milk yield increased with the onset of lactation, and it was higher in the SCHK and SCHC goats at 1-week post-kidding in comparison to that in HEAL goats (*P* < 0.05), whereas it was significantly lower at 2- (*P* < 0.05) and 3-weeks (*P* < 0.01) postpartum in the SCHK and SCHC groups than control group, respectively (Fig. 1B).

### Changes in plasma BHBA, NEFA, and Ca concentrations

**Fig. 2:**
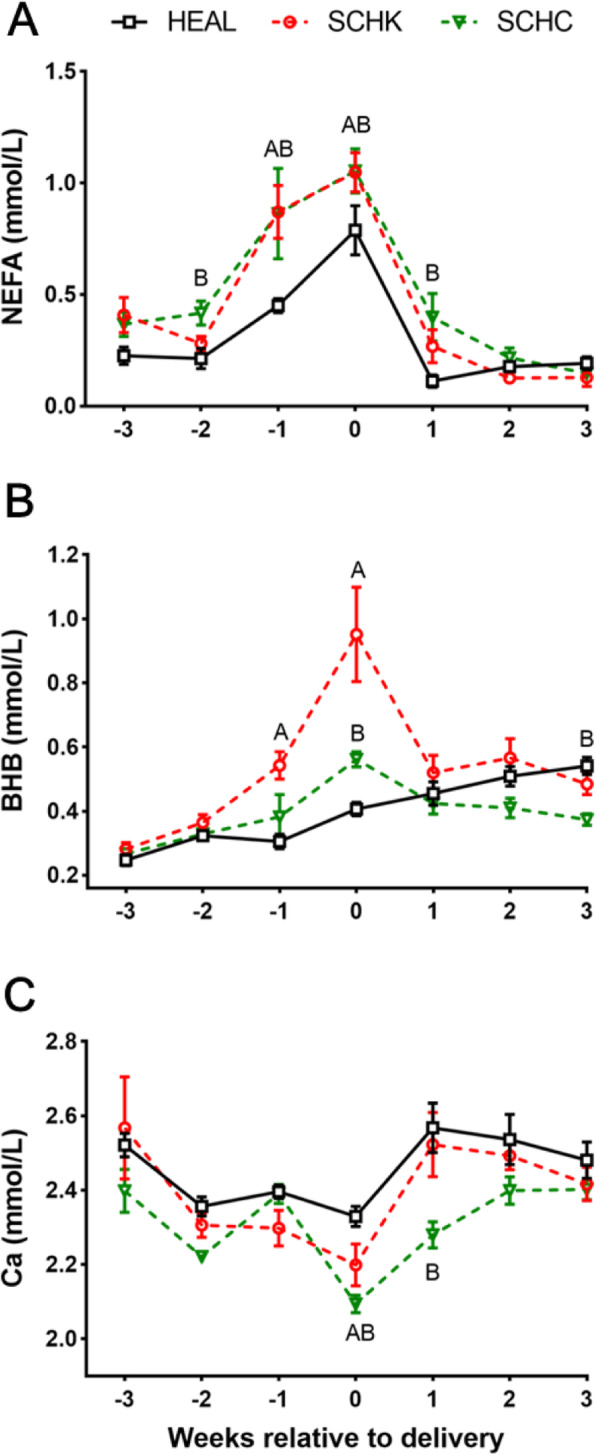
Changes in the concentrations of NEFA (**A**), BHBA (**B**), and Ca (**C**) in the HEAL, SCHK, and SCHC goats during the peripartum period. Plasma biomarkers of metabolites were analyzed by repeated measurements ANOVA, followed by Tukey’s multiple comparisons test. Repeated measures on each goat were considered (repeated factor: time during the periparturient period). ^A^*P* < 0.01 and ^a^*P* < 0.05 between the SCHK and HEAL groups. ^B^*P* < 0.01 and ^b^*P* < 0.05 between the SCHC and HEAL groups. Values are expressed as means ± SEM from 10 goats/group (*n* = 10)

As compared with those for the HEAL goats, the plasma concentrations of non-esterified fatty acids (NEFA) and β-hydroxybutyrate (BHBA) were higher for the SCHK goats at 1-week antepartum and at the day of parturition (*P* < 0.01; Fig. 2 A, B). The plasma NEFA concentrations were significantly greater in the SCHC group from 2-week antepartum to 1-week postpartum (*P* < 0.01; Fig. 2 A), and the BHBA concentrations were significantly greater at parturition (*P* < 0.01; Fig. 2B). The plasma calcium (Ca) concentrations were significantly lower on the day of parturition in the SCHK and SCHC goats than in the HEAL goats (*P* < 0.01) and they remained significantly lower in the SCHC group at 1-week postpartum (*P* < 0.01, Fig. 2 C).

### Changes in the plasma oxidative/antioxidative status indicators

**Fig. 3:**
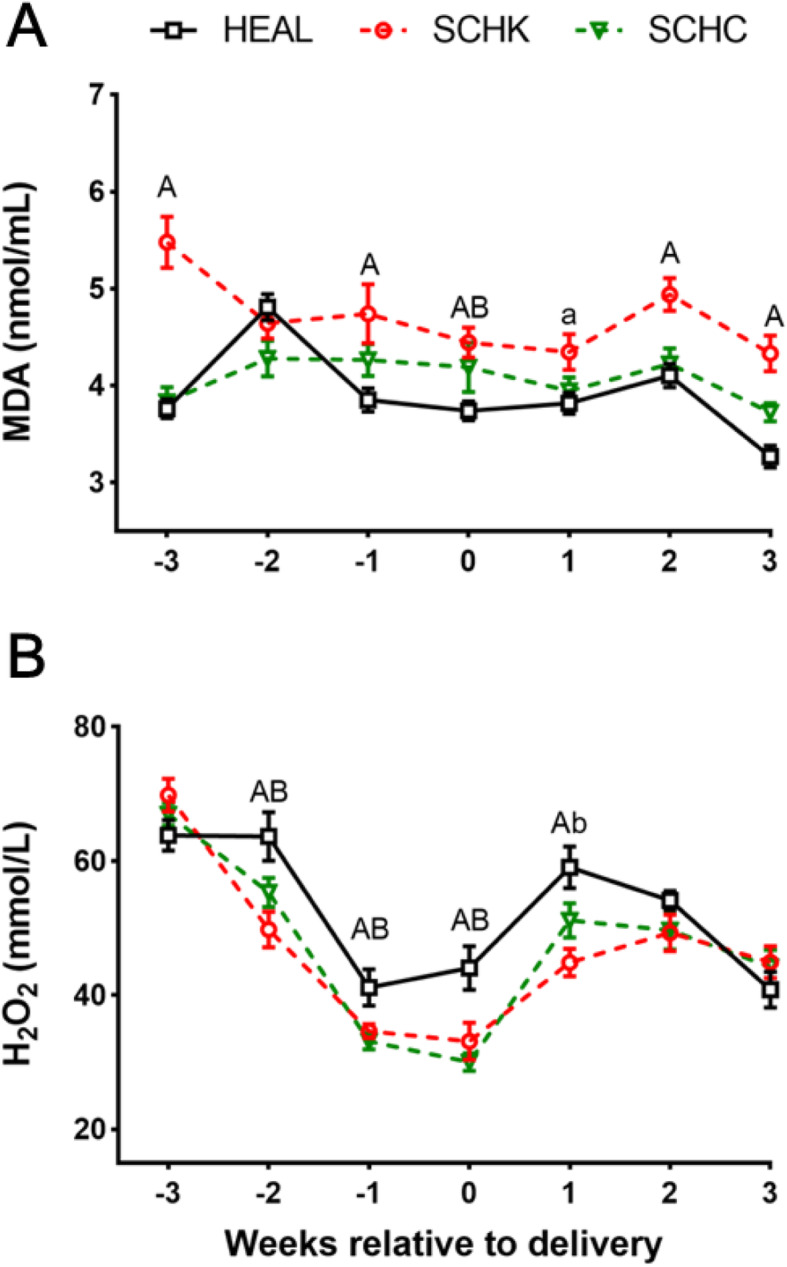
Changes in the MDA (**A**) and H_2_O_2_ (**B**) concentrations in HEAL, SCHK, and SCHC goats during the peripartum period. The plasma MDA and H_2_O_2_ concentrations were analyzed by repeated measurements ANOVA followed by Tukey’s multiple comparisons test. Repeated measures on each goat were considered (repeated factor: time during the periparturient period). ^A^*P* < 0.01 and ^a^*P* < 0.05 between the SCHK and HEAL groups. ^B^*P* < 0.01 and ^b^*P* < 0.05 between the SCHC and HEAL groups. Values are expressed as means ± SEM from 10 goats/group (*n* = 10)

As compared with that in the HEAL goats, the plasma MDA concentration was greater in the SCHK goats from 1-week antepartum to 3-week postpartum (*P* < 0.05 or *P* < 0.01), and it was greater in the SCHC goats at parturition (*P* < 0.01; Fig. 3 A). Conversely, the plasma H_2_O_2_ concentrations were significantly lower in the SCHK and SCHC groups from late pregnancy (2-week antepartum) to early lactation (1-week postpartum) (*P* < 0.05 or *P* < 0.01; Fig. 3B).

**Fig. 4:**
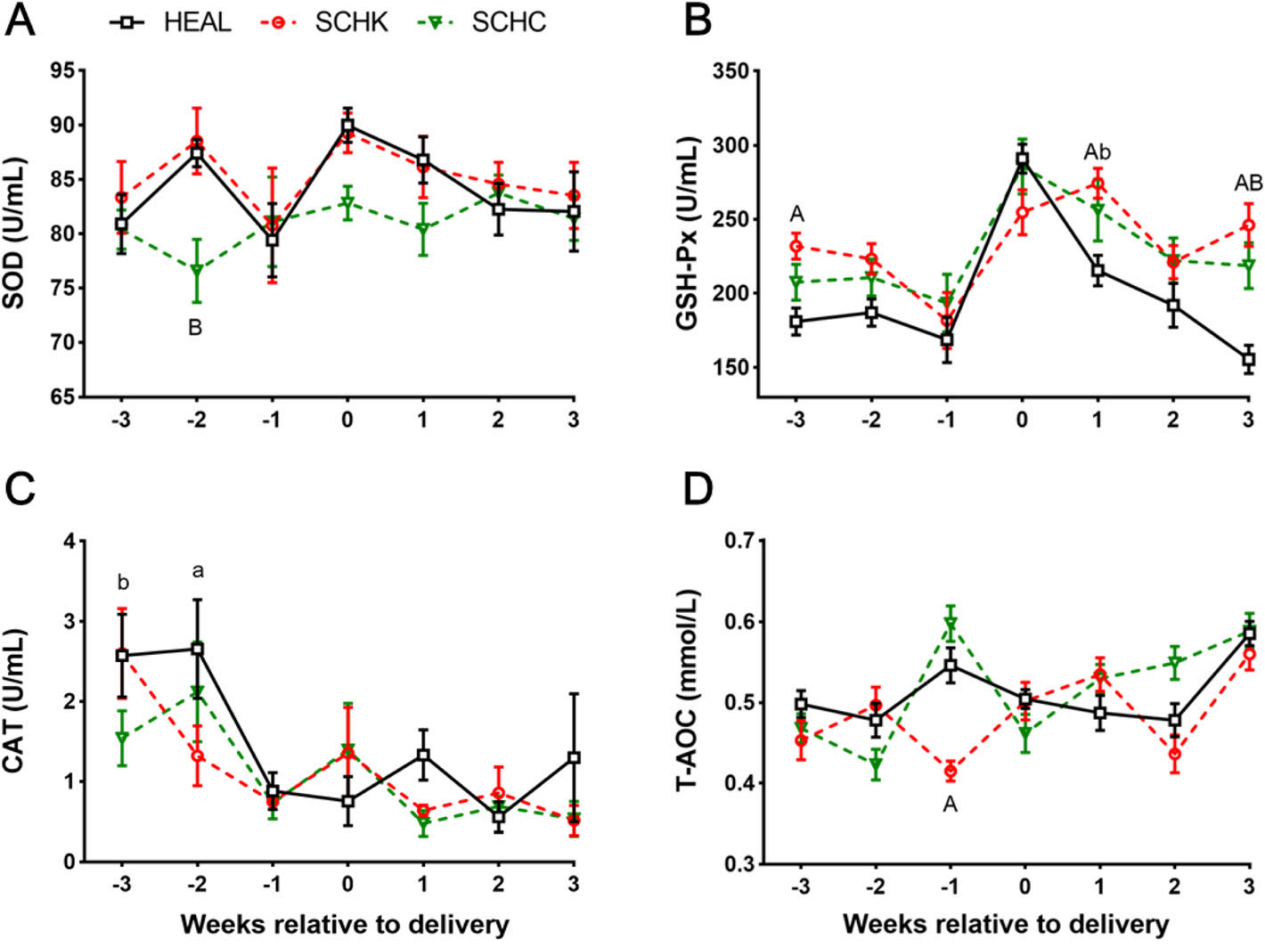
Changes in plasma antioxidant enzymes and total antioxidant capacity in the HEAL, SCHK, and SCHC goats during the peripartum period. (**A**) SOD activity; (**B**) SGH-Px activity; (**C**) CAT activity; (**D**) T-AOC content. The plasma variables were analyzed by repeated measurements ANOVA, followed by Tukey’s multiple comparisons test. Repeated measures on each goat were considered (repeated factor: time during the periparturient period). ^A^*P* < 0.01 and ^a^*P* < 0.05 between the SCHK and HEAL groups. ^B^*P* < 0.01 and ^b^*P* < 0.05 between the SCHC and HEAL groups. Values are expressed as means ± SEM from 10 goats/group (*n* = 10)

The activity of superoxide dismutase (SOD) was lower in the SCHC goats at 2-week antepartum (*P* < 0.01) and did not differ between the SCHK and HEAL goats (Fig. 4 A). The activity of glutathione peroxidase (GSH-Px) was greater in the SCHK and SCHC groups during the early lactation period (at 1- and 3-week postpartum) than that in the HEAL group (*P* < 0.05 or *P* < 0.01; Fig. 4B). The catalase (CAT) concentration was lower in the SCHK and SCHC goats at 2- and 3-week antepartum, respectively (*P* < 0.05; Fig. 4 C). The total antioxidant capacity (T-AOC) was significantly lower at 1-week antepartum in the SCHK group than in the HEAL group (*P* < 0.01; Fig. 4D). No difference was noted in the T-AOC between the SCKC and HEAL groups.

**Fig. 5:**
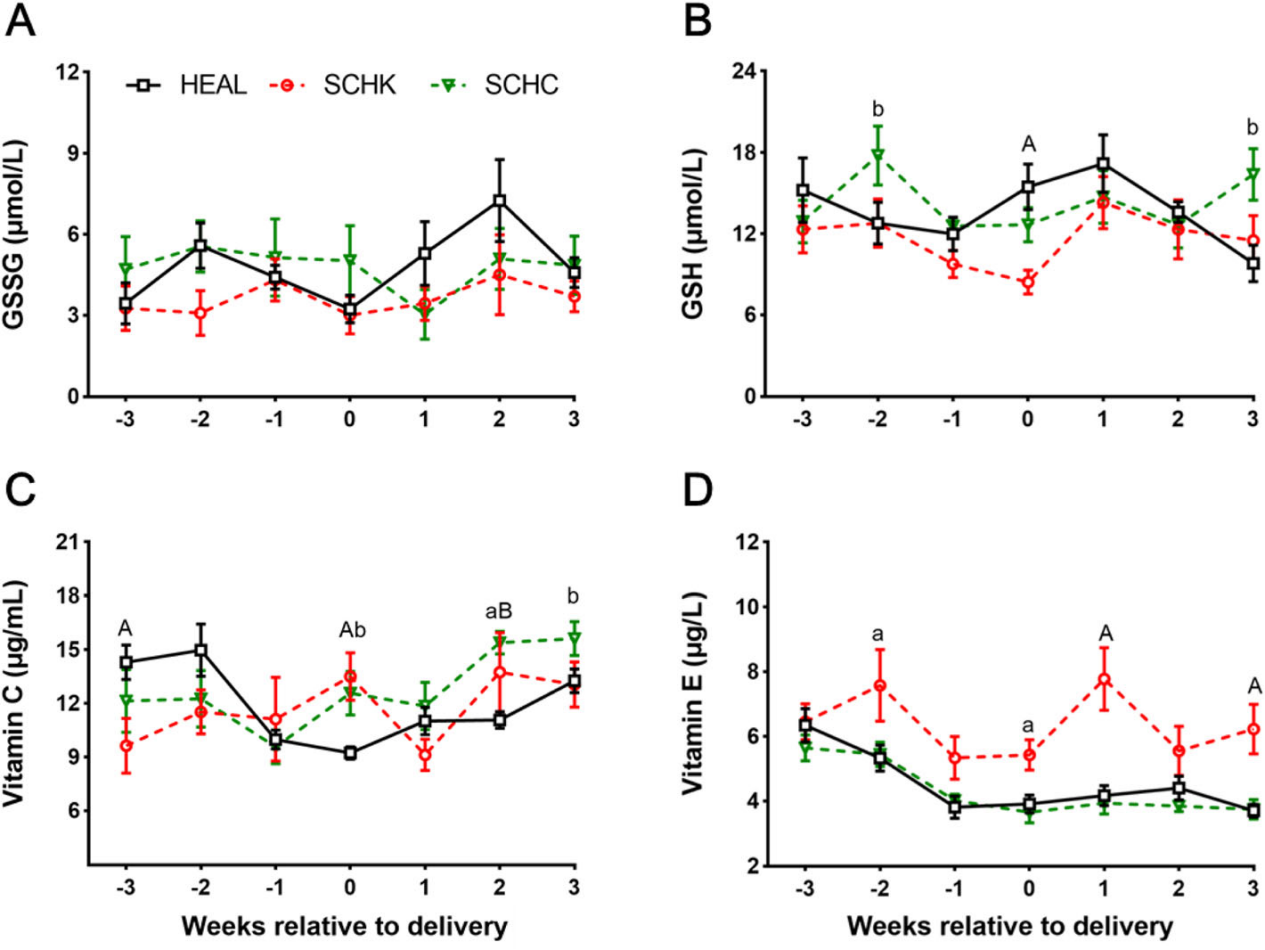
Changes in the plasma antioxidant indexes in the HEAL, SCHK, and SCHC goats during the peripartum period. (**A**) GSH concentration; (**B**) GSSG concentration; (**C**) VC concentration; (**D**) VE concentration. The plasma variables were analyzed by repeated measurements ANOVA, followed by Tukey’s multiple comparisons test. Repeated measures on each goat were considered (repeated factor: time during the periparturient period). ^A^*P* < 0.01 and ^a^*P* < 0.05 between the SCHK and HEAL groups. ^B^*P* < 0.01 and ^b^*P* < 0.05 between the SCHC and HEAL groups. Values are expressed as means ± SEM from 10 goats/group (n = 10)

As compared with the HEAL group, the SCHC group showed higher plasma glutathione (GSH) concentration at 2-week antepartum and 3-week postpartum (*P* < 0.05), while the SCHK group showed lower plasma GSH level at parturition (*P* < 0.01; Fig. 5 A). No significant difference was noted in the plasma glutathione disulfide (GSSG) level (Fig. 5B). A higher plasma vitamin C (VC) concentration was recorded at parturition and at 2-week postpartum in the SCHK and SCHC groups than that in the HEAL group (*P* < 0.05 or *P* < 0.01; Fig. 5 C). As compared with the HEAL group, the SCHK group showed higher plasma vitamin E (VE) concentrations from 2-week antepartum to 3-week postpartum (*P* < 0.05 or *P* < 0.01), which did not differ between the SCHC and HEAL groups (Fig. 5D).

## Discussion

In dairy goats, the periparturient period is especially critical as it presents considerable physiological challenges to the regulation of homeostasis owing to the presence of various metabolic stressors that could drive the onset of diverse disorders [21, 23]. Blood NEFA is one of the most essential indicators of energy balance status [24], and changes in the NEFA concentrations reflect the fat mobilization rate from storage [25]. The mobilizing of adipose tissues is common and a physiological process that occurs around the time of parturition. However, the high plasma NEFA concentrations have been reported to impair the capacity of complete oxidation of fatty acids in the liver, which in turn induce an increase in the levels of free radicals [26]. A previous study reported a blood NEFA concentrations of 0.20–0.22 mmol/L as zero energy balance in lactating goats [27]. In our study, we noted that the HEAL goats experienced moderate lipomobilisation during this period. On the other hand, the dairy goats with SCHK and SCHC suffered relatively more intense lipomobilisation than HEAL goats due to being energy deficit during the peripartal period. The dairy goats with SCHK and SCHC showed more marked decrease in the BCS after kidding, and their milk yields were lower during the early lactation period, except at 1-week postpartum. These data suggest that the influences of hyperketonemia and hypocalcemia on a goat’s production performance may remain over a long term, although these clinicopathological phenomena occurred temporarily at parturition in the present study.

As a significant underlying factor, oxidative stress can induce several metabolic dysfunctions, particularly under increased metabolic stress conditions [4, 9, 28, 29]. Studies have shown that goats experienced oxidative stress during the peripartal period [21, 23]. Importantly, the oxidative status is affected by metabolic adaptation as well [30]. Under energy-deficit conditions, NEFA enters into the hepatocellular mitochondria, where it is oxidized to produce energy. Meanwhile, the increased rate of NEFA oxidation generates excessive reactive oxygen species (ROS), for example, H_2_O_2_ induction promotes the lipoperoxidative processes and changes the redox status [31]. The rise of MDA and H_2_O_2_ may reflect a disturbance in the antioxidant defense mechanisms [32, 33]. Previous studies have shown that, during the dry period, the blood MDA concentrations get elevated in dairy cows [30]. A similar pattern was observed in our study. For instance, in HEAL goats, the increased concentrations of plasma MDA in the late pregnancy (at 2-week antepartum) resulted from excessive lipid peroxidation during this period.

The enzymatic antioxidants including SOD, GSH-Px, and CAT act as the first defense against formation of free radicals by catalyzing their conversion to less reactive or inert species [34, 35]. In this study, the changes in the antioxidant enzymatic activities (SOD, GSH-Px) were consistent with those reported by Radin et al., further indicating that the dairy goats in their late pregnancy and early lactation may have experienced some degree of lipid peroxidation [22]. However, Celi et al. revealed that the blood GSH-Px activity was not significantly different on days − 3, 1, 14, and 28 from parturition when compared with that on day − 21 [23]. The possible reasons for this contradiction may be the difference in the parity of dairy goats. Radin et al. reported that the changes in the oxidative status were closely associated to parity in dairy goats during the transition period [22]. In the present study, the plasma GSH-Px level was sharply increased at parturition when compared with that at 1-week antepartum. This finding was consistent with that of the study of Radin et al. [22] on blood GSH-Px of primiparous goats, as they also found no significant fluctuation in this factor of multiparous goats. Although parity has not been mentioned in the study by Celi et al., we believe that they assessed multiparous goats considering the mention of using 4-5-year-old animals [23].

The antioxidants of non-enzymatic molecules also play an important role. In the presence of GSH, H_2_O_2_ can be decomposed into water and molecular oxygen together with GSH-PX and GSSG reductase [36]. A previous study showed that the GSH level reached its lowest at 1-week postpartum [37]. However, in our experiments, the plasma GSH levels decreased before and after the delivery, while the plasma GSSG levels increased during the prenatal and postpartum periods. Interestingly, the GSSG changes were always accompanied by GSH and were delayed by 1 week. The increase in GSSG level after delivery also reflected the oxidative status of the goat during the early lactation period. Vitamins C and E are non-enzymatic antioxidants that can reduce and, thereby neutralize, the ROS such as H_2_O_2_ [38, 39]. As a primary lipid-soluble and chain-breaking antioxidant, VE protects the integrity of membranes via direct reaction of free radicals [40]. Similarly, as a water-soluble antioxidant, VC serves as a cofactor for several oxygenases. In addition, it regenerates VE to resist oxidative stress [36]. In this study, decreased concentrations of plasma VC and VE in dairy goats during the transition period, particularly at parturition, suggested that VC and VE are useful in resisting oxidative stress. Combined with the results for changes in the levels of enzymatic and non-enzymatic antioxidants, the decreased plasma H_2_O_2_ concentrations in HEAL goats at 1-week antepartum and at parturition can be explained by several factors, as follows: (i) reduced endogenous production of H_2_O_2_ could result from the decreased SOD activity in the late pregnancy, especially at 1-week antepartum; (ii) H_2_O_2_ was scavenged due to the increased CAT activity at 3- and 2-week antepartum; and (iii) low concentrations of H_2_O_2_ at parturition were related to the increased activity of GSH-Px during the same period. These results demonstrate that the HEAL goats experienced some variations in their oxidative status during the late pregnancy and early lactation periods, mediated by MDA and H_2_O_2_ at antepartum and by the high concentrations of H_2_O_2_ at postpartum, respectively.

In this study, SCHK and SCHC goats showed higher concentrations of NEFA at parturition. The high plasma NEFA concentrations have been reported to impair the capacity of complete oxidation of fatty acids in the liver, which causes an increase in the production of free radicals [26]. In dairy cows, oxidative stress could participate in the pathophysiology of multiple metabolic diseases including ketosis and fatty liver [4, 9, 29]. Bernabucci et al. demonstrated that cows with higher NEFA and BHBA showed lower levels of antioxidants [9]. Furthermore, hyperketonemia and lipid peroxidation have been reported in sheep, suggesting that ketonemia is a risk factor for lipid peroxidation and oxidative stress in ewes affected with pregnancy toxemia [28]. The T-AOC is an integrated parameter for the measurement of the cumulative action of all antioxidants. Our results indicate that, in comparison with HEAL goats, the SCHK goats showed significantly decreased T-AOC at 1-week antepartum. An *in vitro* study by Li et al. [41] revealed that high concentration of BHBA treatment reduced the T-AOC in bovine endometrial cells. Furthermore, NEFA could also reduce the T-AOC content in bovine hepatocytes [42]. However, Ozarda et al. [43] reported that the serum NEFA concentrations were positively correlated to T-AOC in the early neonatal life. If the effect of NEFA and BHBA on the T-AOC is similar to that in dairy cows, it is possible that T-AOC reduction is influenced by the combination of NEFA and BHBA at 1-week antepartum in dairy goats with SCHK, considering that NEFA and BHBA were significantly increased during this period. However, this theory does not explain why T-AOC was increased with the elevation of NEFA and BHBA from 1-week antepartum to the time of parturition in SCHK goats. We assume that the influence of NEFA on T-AOC differs among species. Further researches are warranted to explore the effects of NEFA and BHBA on the oxidation status and the underlying mechanisms behind these events in dairy goats.

The plasma MDA concentrations were greatly increased in SCHK and SCHC goats during the peripartum period, especially at parturition. Similar findings have also been reported by studies on other subclinical ketotic animals [20, 44, 45]. Increased MDA concentrations can be attributed to the elevated level of extramitochondrial oxidation of fatty acids, which in turn contributes to oxidative stress [20]. However, the plasma H_2_O_2_ concentrations were significantly decreased in the SCHK and SCHC goats from the period of late pregnancy (2-week antepartum) to that of early lactation (1-week postpartum) when compared to that in HEAL goats. No significant difference was noted in the SOD activity between the SCHK and HEAL goats throughout the perinatal period, indicating that the generation of H_2_O_2_ may not be different between these 2 groups. However, as compared with that in the HEAL goats, GSH-Px was significantly greater at 3-week prepartum and at early lactation in the SCHK goats. These results cumulatively indicate that the decreased H_2_O_2_ level can be attributed to its removal by GSH-Px in the period from the late pregnancy to early lactation in SCHK goats. The SOD activity was lower during late pregnancy to early lactation period in SCHC goats as compared to that in the HEAL goats, especially at 2-week prepartum, suggesting the reduced production of H_2_O_2_. Moreover, the GSH-Px level was significantly higher at early lactation in SCHC goats, indicating that the reduction of H_2_O_2_ occurred as a co-effect of SOD reduction and GSH-Px increment. In addition, a past research asserts that hypocalcemia can increase parathyroid hormone (PTH) secretion via a negative feedback loop, which results in oxidative stress [46]. Our results showed that, to some extent, SCHC goats are oxidatively stressed. As the secretion of calcitropic hormones was not determined in this study, the notion that these hormones participate in the pathological processes of dairy goats with hypocalcemia by influencing the redox status warrants further exploration.

## Conclusions

The data obtained from the present study provide insight into alterations of metabolic parameters and oxidative status-related indexes in the plasma of dairy goats during the periparturient period. Our results clearly demonstrated the need for monitoring dairy goats during this period, especially when presenting with severe metabolic conditions. As compared with the HEAL goats, the dairy goats with SCHK and SCHC exerted more efforts to maintain their redox homeostasis during the late pregnancy to early lactation period, probably owing to more intense fat mobilization and lipid peroxidation. Against the background of the rapid development of intensive dairy goat farming in China, these results provide new point for prevention and treatment of SCHK and SCHC with high prevalence. Future research should determine the cut-point of the antioxidants and oxidants at the different time points in the blood during the periparturient period as well as examine the relationship between the redox status and the pathogenesis of SCHK and SCHC in dairy goats.

## Methods

### Animals, location, and study design

The present study was conducted in Western China (106°55′57″E, 34°48′41″N) at the experimental farm of the Northwest A&F University (Shaanxi Province, China) during January-March 2019. During the experiment, the environmental temperature and relative humidity were recorded by using a portable device (HN-EHBN; Chino, Japan). In the present study, the mean environmental temperature was 11℃ ± 2℃, while the mean relative humidity was 57 % ± 4 %. Guanzhong dairy goats served as experimental animals. The Guanzhong dairy goat is a unique Chinese dairy goat breed that is widely distributed across the Shaanxi province of China. Its milk yields range from 450 to 600 kg over a lactation period of 7–9 months.

**Fig. 6:**
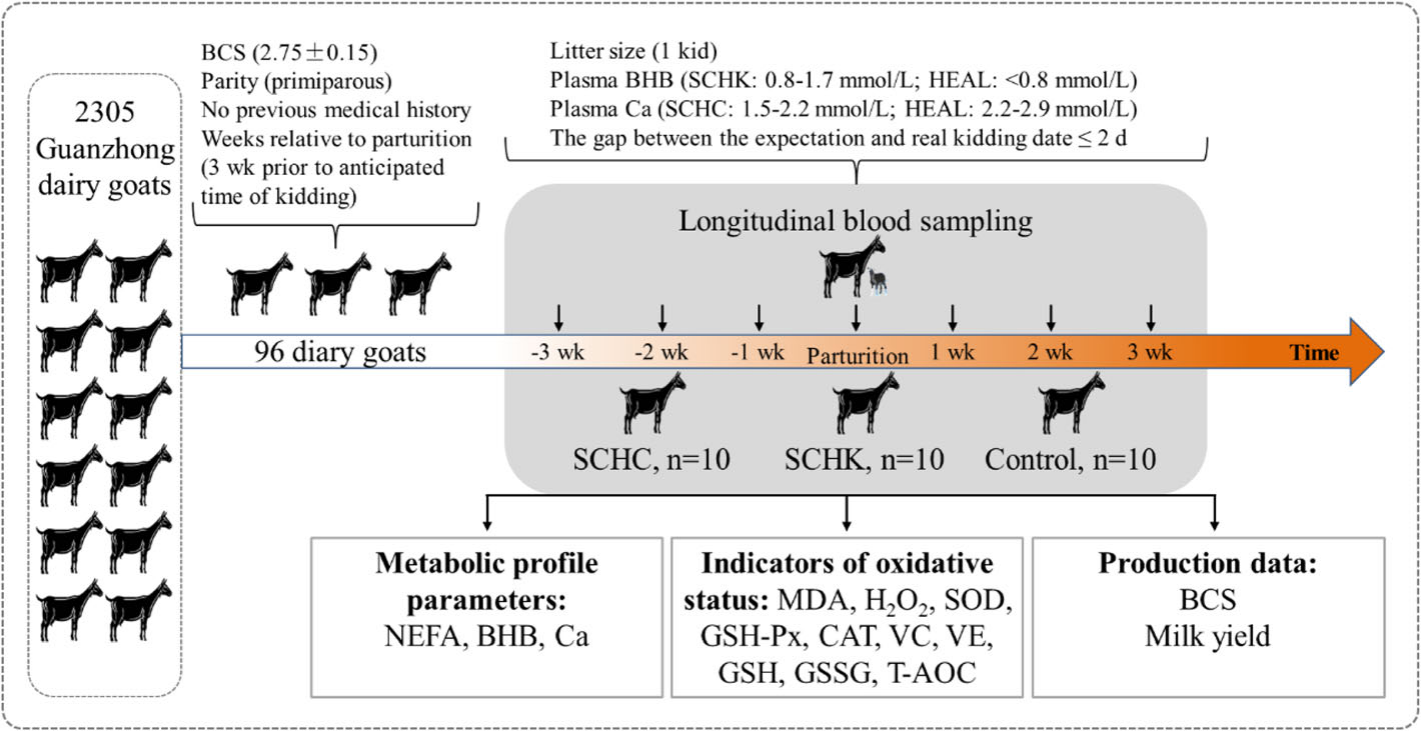
Schematic representation of the study design. The screening and grouping of the experimental goats in 2 steps. (i) 96 primiparous dairy goats randomly selected from 2305 Guanzhong dairy goats based on their similarities in expected parturition date, body condition scores (BCS), and no previous medical history formed the experimental group. The blood samples from each goat were removed at -3, -2, -1, 0 (partum), + 1, +2, and + 3 weeks from delivery. The does were blood sampled within 24 h of their kidding. On the same day of blood sampling, BCS was evaluated, always by the same operator, and the milk yield was recorded for each goat. (ii) Next, in the present study, the criterion for subclinical hyperketonemia (SCHK) and subclinical hypocalcemia (SCHC) in dairy goat were blood BHBA level of 0.8–1.7 mmol/L and Ca level of 1.5–2.2 mmol/L at parturition, respectively. Based on the blood analyses, litter size (1 kid) and the gap between the expected and actual kidding dates (≤ 2 days), 10 goats with high BHBA level and 10 goats with low Ca level at parturition were randomly selected to form the SCHK and SCHC groups, respectively. In addition, 10 goats with 2.2–2.9 mmol/L Ca level and < 0.8 mmol/L BHBA level during the trial period and no signs of other diseases were randomly selected to form the healthy (HEAL) group. Finally, plasma biomarkers of oxidative status and metabolites were enumerated

The experimental protocol adopted in this study were approved by the Ethics Committee on the Use and Care of Animals at Northwest A&F University (Yangling, China) and were conducted in accordance with the university’s guidelines for animal research. Every effort was made to minimize the pain and suffering of animals, and also to decrease the number of animals used. At the end of the study, all animals were released. The present study protocol is schematically presented in Fig. 6. The screening and grouping of experimental goats were performed in 2 steps: i) 96 non-lactating primiparous goats in their late pregnancies were randomly selected from 2305 Guanzhong dairy goats at the Qinyang Dairy Farm, Shaanxi Province, China. Notably, in September, the farm goats included in this study were estrous synchronized such that kidding occurred in February. The selected criterion was based on the similarities in the expected parturition date (within the first week of February), BCS (2.75 ± 0.15, mean ± SEM), and no previous medical history (based on the veterinary record) to form the experimental group. From the 96 goats, 89 goats with the same litter size (1 kid) were included, while 7 goats were excluded for their litter size being 2 or 3.

ii) In this study, the criterions for SCHK and SCHC in dairy goat were 0.8–1.7 mmol/L blood BHBA and 1.5–2.2 mmol/L Ca, respectively [1, 47]. According to the blood analyses, 14 goats with high BHBA concentrations and 22 goats with low Ca concentrations were included in this study. Finally, considering the gap between the expected and real kidding date (≤ 2 days), 10 goats with high BHBA concentrations and 10 goats with low Ca concentrations at parturition with no signs of clinical hyperketonemia and clinical hypocalcemia were randomly selected as the SCHK and SCHC group goats, respectively. In addition, 10 goats with 2.2–2.9 mmol/L Ca concentrations and BHBA < 0.8 mmol/L during the trial period showed no signs of any disease and were hence assigned to the HEAL group.

The goats were reared in a free-stall barn from 3-weeks prior to the anticipated kidding time until 3 weeks post-kidding. All goats were fed the same diets consisting of a base ration fed as a TMR to allow *ad libitum* consumption. This diet was offered twice daily at 0730 and 1530. The diets were formulated to meet the nutrient requirements of dairy goats with reference to the Nutrient Requirements of Small Ruminants (National Research Council, 2007). The ingredients of the diets are listed in Table 1. The goats had free access to water during the experimental period.

**Table 1:** Ingredients and chemical composition of the antepartum and postpartum diets on a dry matter (DM) basis

Ingredient (% of DM)	Antepartum	Postpartum	Nutrient composition	Antepartum	Postpartum
Alfalfa hay	15.36	18.42	DM (% of fresh)	45.10	48.20
Corn	20.72	23.16	Neutral detergent fiber (NDF, %)	42.20	37.70
Wheat bran	7.20	8.37	Acid detergent fiber (ADF, %)	18.50	17.40
Soybean meal	4.89	9.02	Crude protein (CP, %)	14.20	16.40
Wheat straw	7.57	0.00	Starch (%)	24.3	25.40
Corn Silage	35.35	30.66	Ether extract (%)	3.00	3.20
Corn germ meal	2.80	3.26	Calcium (Ca, %)	0.48	0.66
Cottonseed meal	4.40	5.12	Phosphorus (P, %)	0.36	0.37
Calcium hydrophosphate	0.44	0.51	Magnesium (Mg, %)	0.14	0.19
Limestone	0.40	0.46	Sulfur (S, %)	0.20	0.20
Sodium carbonate	0.32	0.38	Chloride (Cl, %)	0.47	0.25
Sodium chloride	0.40	0.45	DCAD (mEq/kg of DM) ^b^	-164	+ 733
Mineral and vitamin premix ^a^	0.15	0.19	NE_L_ (Mcal/kg) ^c^	1.53	1.62

^a^ The mineral-vitamin premix provided the following per kg of diets: vitamin A 250,000 IU, vitamin D 23,250 IU, vitamin E 1500 IU, manganese 800 mg, zinc 1800 mg, copper 370 mg, iron 2200 mg, cobalt 50 mg, iodine 30 mg, selenium 30 mg.

^b^ The dietary cation-anion difference (DCAD) was calculated using the formula DCAD (mEq/kg of DM) = (Na^+^ + K^+^) − (Cl^−^ + S^2−^).

^c^ Net energy for lactation (NE_L_) was estimated using CPM-Dairy software (version 3.0.8.1).

### Production performance, plasma samples, and laboratory analyses

The blood samples (10 mL) were collected from individual goats at -3, -2, -1, 0 (partum), + 1, +2, and + 3 weeks from delivery from the jugular vein using vacutainer tubes containing sodium heparin (Becton-Dickinson, Franklin Lakes, NJ). At antepartum, the blood samples for each goat were collected before feeding in the morning. At partum (0), the blood of the does were sampled within 24 h of kidding. At postpartum, the blood samples were collected after the milking operations, but before feeding in the morning. All the tubes for plasma collection were immediately placed on an ice bath. The blood samples, collected within 1 h of bleeding, were centrifuged at 2000 ×*g* for 10 min; and the supernatant plasma was packaged and stored at -80 °C until further use. The biomarkers of oxidative status and metabolites were measured within 2 weeks of bleeding. On the same day of blood sampling, BCS was evaluated, always by the same operator, following the method proposed by Santucci et al. [48], and the milk yield was recorded for each goat (starting from the first week of lactation). Notably, based on the feeding scheme of the kids in the farm, the does were not milked when feeding the kids freely within the first week of lactation. Later, the kids were separated from the does and artificial suckling was performed. From the first week of lactation, the goats were milked using a bucket milking machine, twice a day (at 0630 and 1430).

The plasma BHBA (kit no. RB1007, enzymatic method), NEFA (kit no. FA115, colorimetric method), and Ca (kit no. CA590, Arsenazo III method) concentrations were analyzed by using commercial kits (Randox Laboratories, Crumlin, UK) and a Hitachi auto-analyzer (Hitachi, Fukuoka, Japan).

The plasma concentrations of MDA (kit no. A003-1), H_2_O_2_ (kit no. A064-1), VC (kit no. A009-1), GSH (kit no. A006-2), GSSG (kit no. A061-1), and T-AOC (kit no. A015-2) were measured as per the manufacturer’s instructions using corresponding commercial kits (Nanjing Jiancheng Bioengineering Institute, China). MDA was measured by the TBA method. The measured intra- and interassay coefficients of variation (CV) were 7.6 and 6.3 %, respectively. The levels of H_2_O_2_, VC, GSH, and GSSG were measured by colorimetric method. The intra- and interassay CV of H_2_O_2_ were 8.8 and 7.9 %, those of VC were 8.6 and 5.2 %, those of GSH were 9.6 and 6.8 %, and those of GSSG were 7.2 and 7.5 %, respectively. The plasma concentrations of T-AOC were measured by the ABTS method. The intra- and interassay CV of T-AOC were 8.1 and 7.2 %, respectively.

Commercial kits were used to determine the enzymatic activities of GSH-Px, SOD, and CAT (Nanjing Jiancheng Bioengineering Institute). The GSH-Px level was determined in the plasma by colorimetric method (kit no. A005-1). The intra- and interassay CV of GSH-Px were 5.2 and 5.8 %, respectively. SOD was determined using hydroxylamine method (kit no. A001-1). The intra- and interassay CV of SOD were 9.5 and 8.1 %, respectively. CAT was determined by the ammonium molybdate method (kit no. A007-1). The intra- and interassay CV of CAT were 8.9 and 7.8 %, respectively. Commercially available ELISA kits were used to measure the concentrations of VE in the plasma (kit no. MM2719, Meimian Biotechnology, Yancheng, Jiangsu, China). A standard curve was plotted at concentrations from 0.25 to 8.50 µg/L. The intra- and interassay CV of VE were 5.5 and 6.2, respectively.

The absorbance values of MDA, H_2_O_2_, VC, GSH, GSSG, T-AOC, GSH-Px, SOD, CAT, and VE were estimated using the Bio-Rad 680 microplate reader (USA) at 532, 405, 536, 405, 405, 405, 412, 550, 405, and 450 nm, respectively. The results were calculated according to the relevant corresponding formulas.

### Statistical analysis

Statistical analysis of the data was performed by using the GraphPad Prism 7.0 (GraphPad Software Inc., USA). The changes in milk yield, BCS, and plasma biomarkers of oxidative status and metabolites were analyzed using repeated measurements ANOVA, followed by Tukey’s multiple comparisons test. Repeated measures on each goat were considered (repeated factor: time during the periparturient period). The results were expressed as mean ± standard error of the mean (SEM).

## Data Availability

The datasets used and/or analysed during the present study available from the corresponding author on reasonable request.

## Abbreviations

SCHK: Subclinical hyperketonemia
SCHC: Subclinical hypocalcemia
HEAL: Health
NEFA: Non-esterified fatty acids
BHBA: β-hydroxybutyrate
Ca: Calcium
BCS: Body condition scores
DMI: Dry matter intake
MDA: Malondialdehyde
H_2_O_2_: Hydrogen peroxide
ROS: Reactive oxygen species
ROMs: Reactive oxygen metabolites
T-AOC: Total antioxidant capacity
SOD: Superoxide dismutase
GSH-Px: Glutathione peroxidase
CAT: Catalase
GSH: Glutathione
GSSG: Glutathione disulfide
VE: Vitamin E
VC: Vitamin C
PTH: Parathyroid hormone
